# Functional variants of the *ATG7* gene promoter in acute myocardial infarction

**DOI:** 10.1002/mgg3.508

**Published:** 2018-11-08

**Authors:** Pei Zhang, Jie Zhang, Yexin Zhang, Shuai Wang, Shuchao Pang, Bo Yan

**Affiliations:** ^1^ College of Clinical Medicine Xinxiang Medical University Xinxiang Henan China; ^2^ Division of Emergency Jining First People's Hospital Jining Shandong China; ^3^ Department of Medicine Shandong University School of Medicine Jinan Shandong China; ^4^ Shandong Provincial Key Laboratory of Cardiac Disease Diagnosis and Treatment Affiliated Hospital of Jining Medical University Jining Medical University Jining Shandong China; ^5^ The Center for Molecular Genetics of Cardiovascular Diseases Affiliated Hospital of Jining Medical University Jining Medical University Jining Shandong China; ^6^ Shandong Provincial Sino‐US Cooperation Research Center for Translational Medicine Affiliated Hospital of Jining Medical University Jining Medical University Jining Shandong China

**Keywords:** acute myocardial infarction, ATG7, autophagy, DNA sequence variants, genetics, promoter

## Abstract

**Background:**

Coronary artery disease including acute myocardial infarction (AMI) is mainly caused by atherosclerosis, an inflammatory and metabolic disease. Autophagy has been demonstrated to play critical roles in lipid metabolism and inflammation. Altered autophagic activity has been reported in AMI patients. However, molecular basis for dysfunctional autophagy in AMI remains unexplained.

**Methods:**

In this study, the promoter of the *ATG7* gene, encoding a core protein for autophagy, was genetically and functionally analyzed in large cohorts of AMI patients (*n* = 355) and ethnic‐matched healthy controls (*n* = 363). Related molecular mechanisms were also explored.

**Results:**

A total of 19 DNA sequence variants (DSVs) including single‐nucleotide polymorphisms (SNPs) were found in the *ATG7* gene promoter. Two novel DSVs and five SNPs were only identified in AMI patients group. These DSVs and SNPs, except one SNP, significantly altered the transcriptional activity of the *ATG7* gene promoter in both HEK‐293 and H9c2 cells (*p* < 0.05). Further electrophoretic mobility shift assay revealed that the DSVs and SNPs evidently affected the binding of transcription factors.

**Conclusions:**

*ATG7* gene DSVs and SNPs identified in AMI patients may alter the transcriptional activity of the *ATG7* gene promoter and change ATG7 level, contributing to the AMI development as a rare risk factor.

## 1 INTRODUCTION

Coronary artery disease (CAD) including acute myocardial infarction (AMI) is a common complex disease that is caused by atherosclerosis, an inflammatory and metabolic disease. Dysregulated lipid metabolism and inflammation have been shown to play critical roles in the initiation and progression of atherosclerosis and its complications, particularly plaque rupture and subsequent AMI (Connelly, Shalaurova, & Otvos, 2016; Shapiro & Fazio, 2016). Genome‐wide association studies have revealed a great number of genetic loci for CAD and AMI, some of which are associated with lipid metabolism and inflammation. However, the collective genetic loci could explain only <10% of cases (Assimes & Roberts, 2016). Therefore, genetic causes and molecular mechanisms for CAD and AMI remain largely unclear.

Autophagy is an evolutionally conserved cellular process to maintain cell homeostasis by delivering cytoplasmic contents to lysosomes for degradation. In mammals, autophagy has three subtypes, macroautophagy, microautophagy, and chaperone‐mediated autophagy. Macroautophagy (hereafter referred as autophagy) mainly degrades long‐lived macromolecules and damaged organelles and has been extensively studied. Autophagy is involved in many physiological processes, including lipid metabolism and inflammation. Altered autophagic activity has been implicated in a wide range of human diseases, including cardiovascular diseases (Lavandero, Chiong, Rothermel, & Hill, 2015). In the cardiovascular system, autophagic activity occurs in all cell types including cardiomyocytes, cardiac fibroblasts, vascular smooth muscle cells, vascular endothelial cells, and macrophages. A window of optimal autophagic activity is critical to the maintenance of cardiovascular homeostasis and function. Excessive or insufficient levels of autophagic flux can each contribute to the pathogenesis of cardiovascular diseases, such as atherosclerosis, cardiomyopathy, and AMI (Bravo‐San Pedro, Kroemer, & Galluzzi, 2017; Gatica, Chiong, Lavandero, & Klionsky, 2015; Lavandero et al., 2015).

To date, more than 30 autophagy‐related (ATG) proteins have been identified in the formation of autophagosome (Mizushima, Yoshimori, & Ohsumi, 2011). ATG7 (OMIM: 608760) acts as an E1‐like activating enzyme and facilitates both microtubule‐associated protein light chain 3 (LC3)‐phosphatidylethanolamine and ATG12 conjugation, which play important roles in autophagosome biogenesis in autophagy. ATG7 has been shown to play a pleiotropic function in development and human diseases (Xiong, 2015). Homozygous knockout of *ATG7* gene in mice is neonatally lethal, and autophagy is not upregulated during starvation (Komatsu et al., 2005). Tissue‐specific deletion of *ATG7* gene and subsequent defective autophagy has been widely studied, including cardiomyocytes, vascular endothelial cells, and smooth muscle cells. Conditional knockout of *ATG7* gene in cardiomyocyte leads to severe contractile dysfunction, myofibrillar disarray, and vacuolar cardiomyocytes (Li et al., 2016). In a mouse model of cardiomyopathy, cardiac‐specific overexpression of *ATG7* gene and enhanced autophagy improve cardiac performance by decreasing interstitial fibrosis, ameliorating ventricular dysfunction, decreasing cardiac hypertrophy, and reducing intracellular aggregates (Bhuiyan et al., 2013). In neonatal rat cardiomyocytes, ATG7 induces basal autophagy and attenuates the accumulation of misfolded proteins and aggregates (Pattison, Osinska, & Robbins, 2011). In vascular smooth muscle cells, *ATG7* gene deletion alters contractility and Ca²⁺ homeostasis (Michiels, Fransen, De Munck, De Meyer, & Martinet, 2015). In endothelial cells, ATG7 gene deletion results in higher levels of intracellular lipid accumulation (Torisu et al., 2016).

Dysregulated gene expression has been implicated in many human common diseases (Lee & Young, 2013). In previous studies, we have reported altered autophagic activity in CAD and AMI patients by examining LC3 gene expression levels (Wu, Wei et al., 2011; Wu, Liu et al., 2011). However, molecular basis for dysfunctional autophagy in AMI remains unexplained. As ATG7 is one of core proteins for autophagy, we postulated that dysregulated *ATG7* gene expression may lead to altered autophagic activity, playing an important role in the development of CAD and AMI. In this study, we genetically and functionally analyzed the promoter of *ATG7* gene in large cohort of AMI patients and ethnic‐matched healthy controls.

## 2 MATERIAL AND METHODS

### 2.1 Study population

Acute myocardial infarction patients (*n* = 355, male 265, female 90, median age 63.00 years) were recruited during the period April 2014 to April 2016, from Cardiac Care Unit, Division of Cardiology, Affiliated Hospital of Jining Medical University, Jining, Shandong, China. AMI patients were diagnosed with medical records, physical examination, electrocardiogram, and three‐dimensional echocardiography. Ethnic‐matched healthy controls (*n* = 363, male 188, female 175, median age 51.00 years) were recruited from the Physical Examination Center in the same hospital. The controls with CAD family history were excluded from this study. The study was approved by the Human Ethic Committee of Affiliated Hospital of Jining Medical University and conducted according to the principles of the Declaration of Helsinki. Informed consent was obtained from all individual participants included in the study.

### 2.2 Direct DNA sequencing

Peripheral blood leukocytes were isolated from vein blood, and genomic DNAs were extracted. *ATG7* gene promoter region (from −1145 bp to +112 bp to the transcription start site) was directly sequenced. Two overlapped DNA fragments, 698 bp (−1145 bp ~ −448 bp) and 622 bp (−510 bp~+112 bp), were generated by PCR. PCR primers were designed based on genomic sequence of the human *ATG7* gene (NCBI, NC_000003.12; Table 1). PCR products were bidirectionally sequenced with Applied Biosystems 3500XL genetic analyzer. The DNA sequences were aligned and compared with the wild‐type *ATG7* gene promoter.

**Table 1:** PCR primers for the *ATG7* gene promoter

PCR primers^a^	Sequences	Location	Position^b^ (bp)	Products (bp)
Sequencing				
ATG7‐F1	5′‐GGTTCCTTCTCTCCCACCTC‐3′	11271179	−1145	698
ATG7‐R1	5′‐ACTGGACAGGTGTTGAAG ‐3′	11271876	−448	
ATG7‐F2	5′‐GCCTTACAGGCCAGACAGA‐3′	11271814	−510	622
ATG7‐R2	5′‐CTTACCGCCGCTCAACTT‐3′	11272435	+112	
Functioning				
ATG7‐F	5′‐(KpnI)‐ACGGAGTCTCGCTCTGTCGC‐3′	11271242	−1082	1194
ATG7‐R	5′‐(HindIII)‐CTTACCGCCGCTCAACTTCC ‐3′	11272435	+112	

^a^PCR primers are designed based on the genomic DNA sequence of the *ATG7* gene (NC_000003.12). ^b^The transcription start site (TSS) is at the position of 11272324 (+1).

### 2.3 Functional analysis with dual‐luciferase reporter assay

Wild‐type and variant *ATG7* gene promoters were subcloned into firefly luciferase reporter vector (pGL3‐basic) to construct expression vectors. These expression vectors were then transfected into cultured cells, and dual‐luciferase activities were examined. Briefly, DNA fragments of wild‐type and variant *ATG7* gene promoters (1194 bp, from −1082 bp to +112 bp to the transcription start site) were generated by PCR and inserted into the KpnI and Hind III sites of pGL3‐basic to generate expression vectors. The PCR primers are shown in Table 1. Designated expression vectors were transiently transfected into human embryonic kidney cells (HEK‐293) or rat cardiomyocyte line cells (H9c2). Forty‐eight hours post‐transfection, the cells were collected and lysed. Dual‐luciferase activities were measured using dual‐luciferase reporter assay system on a Promega Glomax 20/20 luminometer. Vector pRL‐TK expressing renilla luciferase was used as an internal control for transfection efficiency. Empty vector pGL3‐basic was used as a negative control. The transcriptional activities of the *ATG7* gene promoters were represented as ratios of firefly luciferase activities over renilla luciferase activities. Transcriptional activity of the wild‐type *ATG7* gene promoter was designed as 100%. All the experiments were repeated three times independently, in triplicate.

### 2.4 Nuclear extracts preparation and electrophoretic mobility shift assay (EMSA)

Nuclear extracts were prepared from HEK‐293 and H9c2 cells using NE‐PER^®^ Nuclear and Cytoplasmic Extraction Reagents (Thermo Scientific, Rockford, IL, USA). Protein concentrations were determined with Bio‐Rad protein assay. EMSA examining DNA–protein interactions was carried out using LightShift^®^ Chemiluminescent EMSA kit (Thermo Scientific, Rockford, IL, USA). DNA‐protein binding reaction was carried out with nuclear extracts (3.0 μg) and double‐stranded biotinylated oligonucleotides (30 bp) containing wild‐type and variant DNA sequence of the *ATG7* gene promoter. After 20 min at room temperature, the reaction mixtures were loaded onto a native 6% polyacrylamide gel in 0.5× TBE and run at 100 V for 1 hr, which were subsequently transferred onto a nylon membrane (Thermo Scientific, Rockford, IL, USA) at 100V for 30 min. The oligonucleotides were cross‐linked to the membrane using the UV Stratalinker 1800 (Stratagene, Santa Clara, CA, USA). The biotin‐labeled oligonucleotides were then detected by chemiluminescence.

### 2.5 Statistical analysis

The quantitative data were represented as mean ± *SEM* and compared by a standard Student's *t* test. DSV frequencies in AMI patients and controls were analyzed and compared with SPSS v13.0. *p* < 0.05 was considered statistically significant.

## 3 RESULTS

### 3.1 The DSVs identified in AMI patients and controls

**Figure 1:**
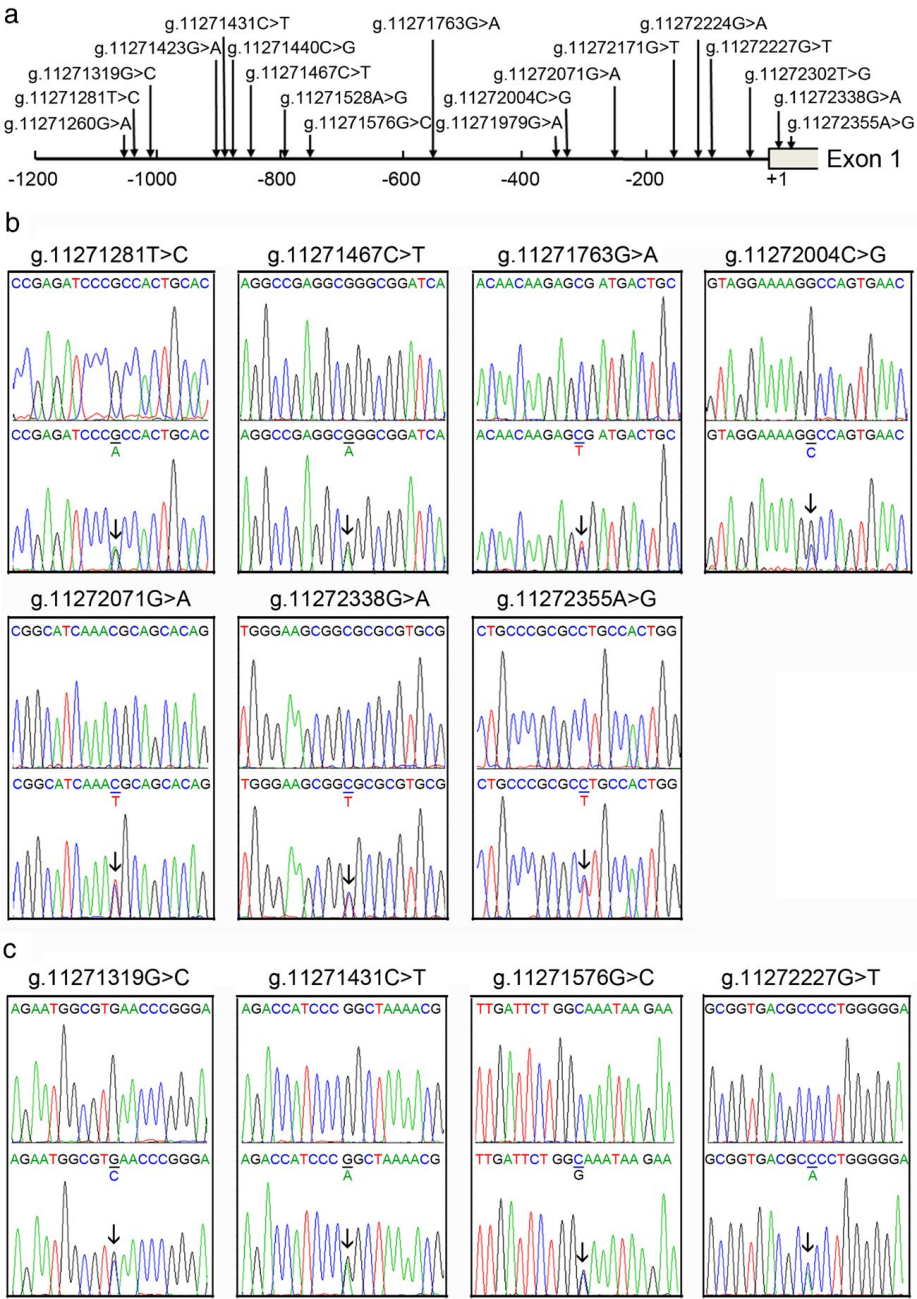
Locations and sequencing chromatograms of the DSVs and SNPs in the *ATG7* gene promoter. (a) Locations of the DSVs and SNPs. The numbers represent the genomic DNA sequences of the human *ATG7* gene (Genbank accession number NC_000003.12) upstream to the transcription start site (at the position of 11272264), which is set as +1. (b) Sequencing chromatograms of the DSVs and SNPs in AMI patients. (c) Sequencing chromatograms of the DSVs in controls. Top panels show wild‐type DNA sequences, and bottom panels heterozygous DSVs and SNPs, which are marked with arrows. For the DSV g.11271319G>C, sequence orientation is forward and all others are reverse

A total of 19 DNA sequence variants (DSVs), including 12 single‐nucleotide polymorphisms (SNPs), were identified in this study population. Locations and frequencies of the DSVs are depicted in Figure 1 and summarized in Table 2. Two novel heterozygous DSVs, g.11271467C>T and g.11272338G>A, and five heterozygous SNPs, g.11271281T>C (rs2594975), g.11271763G>A (rs76708041), g.11272004C>G (rs550744886), g.11272071G>A (rs544484285), and g.11272355A>G (rs2606729), were found in nine AMI patients. In the 1000 Genome Project, frequencies of C allele in g.11271281T>C (rs2594975) and G allele in g.11272355A>G (rs2606729) were 100% in Han Chinese people. These DSVs and SNPs were not found in controls. Sequencing chromatograms of these DSVs and SNPs are shown in Figure 1b. Four novel heterozygous DSVs, g.11271319G>C, g.11271431C>T, g.11271576G>C, and g.11272227G>T, were only identified in controls, the sequencing chromatograms of which are shown in Figure 1c. In addition, one novel heterozygous DSV and seven SNPs were found in both AMI patients and controls with similar frequencies (*p* > 0.05).

**Table 2:** The DSVs in the *ATG7* gene promoter in AMI patients and controls

DSVs	Genotypes	Location[Link] (bp)	Controls (*n* = 363)	AMI (*n* = 355)	*p* value
g.11271260G>A	GA	−1064	1	1	1.000
g.11271281T>C (rs2594975)	TC	−1043	0	2	‐
g.11271319G>C	GC	−1005	1	0	‐
g.11271423G>A (rs533883497)	GA	−901	1	1	1.000
g.11271431C>T	CT	−893	1	0	‐
g.11271440C>G (rs181704508)	CG	−884	26	28	0.778
g.11271467C>T	CT	−857	0	1	‐
g.11271528A>G (rs11714563)	AA	−796	176	165	0.730
	AG		157	163	
	GG		30	27	
g.11271576G>C	GC	−748	1	0	‐
g.11271763G>A (rs76708041)	GA	−561	0	1	‐
g.11271979G>A (rs552163870)	GA	−345	2	5	0.282
g.11272004C>G (rs550744886)	CG	−320	0	2	‐
g.11272071G>A (rs544484285)	GA	−253	0	1	‐
g.11272171G>T (rs140788185)	GT	−153	4	6	0.542
g.11272224G>A (rs2594971)	GG	−100	48	45	0.206
	GA		168	187	
	AA	‐	147	123	
g.11272227G>T	GT	−97	1	0	‐
g.11272302T>G (rs142065270)	TT	−22	359	348	0.477
	TG		4	6	
	GG		0	1	
g.11272338G>A	GA	+15	0	1	‐
g.11272355A>G (rs2606729)	AG	+32	0	1	‐

DSVs are located upstream (‐) or downstream (+) to the transcription start site of ATG7 gene at 11272324 of NC_000003.12.

### 3.2 DSV‐related putative binding sites for transcription factors

To determine whether the DSVs and SNPs identified in AMI patients affected putative binding sites for transcription factors, the *ATG7* gene promoter was analyzed with JASPAR program (http://jaspar.genereg.net/). The DSVs identified in AMI patients may abolish, create, and modify the putative binding sites for transcription factors, which are summarized in Table 3. The SNP g.11271281T>C (rs2594975) may abolish a binding site for zinc finger protein 354C (ZF354C), a ubiquitously expressed transcriptional repressor. The DSV g.11271467C>T may abolish a binding site for THAP domain containing 1 (THAP1), a C2CH THAP‐type zinc finger factor with roles in proliferation, apoptosis, cell cycle, chromosome segregation, chromatin modification, and transcriptional regulation. The SNP g.11271763G>A (rs76708041) may modify the binding site for GATA binding protein 2 (GATA2), a transcriptional activator involved in the development and proliferation of hematopoietic and endocrine cell lineages. The SNP g.11272004C>G (rs550744886) may create the binding sites for Rhox homeobox family member 1 (RHOXF1), a transcription factor that may play a role in human reproduction, and NF‐kappaB‐related factors. The SNP g.11272071G>A (rs544484285) may abolish the binding site for transcription factor 7‐like 2 (TCF7L2), which is highly expressed in fat. The DSV g.11272338G>A may modify the binding site for nuclear respiratory factor 1 (NRF1), which regulates cellular growth and nuclear genes required for respiration, heme biosynthesis, and mitochondrial DNA transcription and replication. The SNP g.11272355A>G (rs2606729) may modify the binding site for nuclear factor 1 X‐type (NFIX), a SMAD/NF‐1 DNA‐binding domain transcriptional activator.

**Table 3:** Predicted binding sites for transcription factors affected by the DSVs and SNPs

DSVs/SNPs	Create/abolish/modify	Transcription factors
g.11271281T>C (rs2594975)	Abolish	zinc finger protein 354C (ZF354C),
g.11271467C>T	Abolish	C2CH THAP‐type zinc finger factor THAP domain containing 1 (THAP1)
g.11271763G>A (rs76708041)	Modify	GATA binding protein 2 (GATA2)
g.11272004C>G (rs550744886)	Create	Rhox homeobox family member 1 (RHOXF1)
g.11272071G>A (rs544484285)	Abolish	transcription factor 7 like 2 (TCF7L2)
g.11272338G>A	Modify	nuclear respiratory factor 1 (NRF1)
g.11272355A>G (rs2606729)	Modify	nuclear factor 1 X‐type (NFIX)

### 3.3 Functional analysis of the DSVs by dual‐luciferase reporter assay

Wild‐type and variant *ATG7* gene promoters were subcloned into luciferase reporter vector (pGL3‐basic) to generate expression vectors, including empty pGL3‐basic (negative control), pGL3‐WT (wild‐type *ATG7* gene promoter), pGL3‐11271281T, pGL3‐11271431T, pGL3‐11271467T, pGL3‐11271576C, pGL3‐11271763A, pGL3‐11272004G, pGL3‐11272071A, pGL3‐11272171T, pGL3‐11272227G>T, pGL3‐11272338A, and pGL3‐11272355A. The expression vectors were transfected into HEK‐293 and H9c2 cells, and dual‐luciferase activities were assayed.

**Figure 2:**
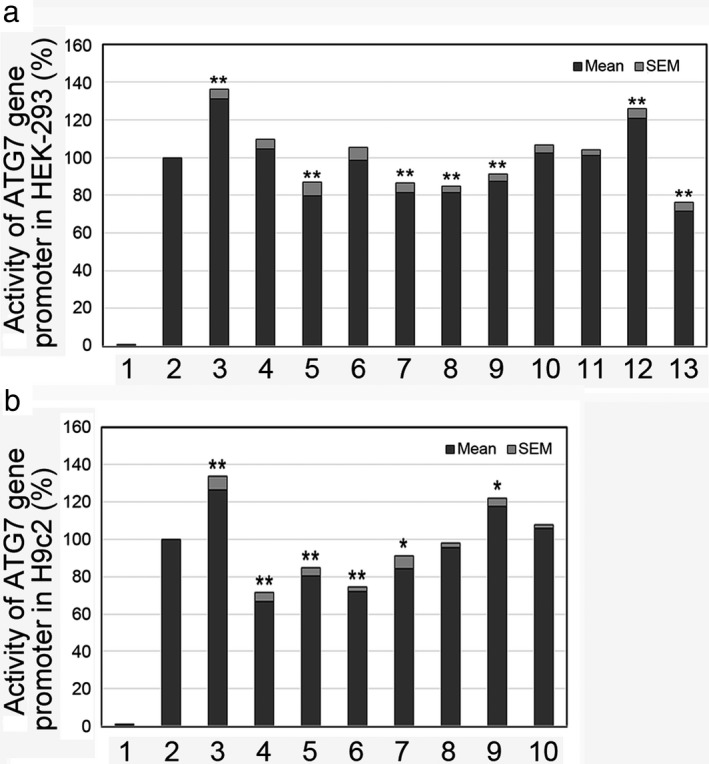
Relative transcriptional activity of wild‐type and variant *ATG7* gene promoters. Wild‐type and variant *ATG7* gene promoters were cloned into reporter gene vector pGL3 and transfected into cultured cells. The transfected cells were collected, and dual‐luciferase activities were assayed. Empty vector pGL3‐basic was used as a negative control. Transcriptional activity of the wild‐type *ATG7* gene promoter was designed as 100%. Relative activities of *ATG7* gene promoters were calculated. (a) Relative activities of wild‐type and variant *ATG7* gene promoters in HEK‐293 cells. Lanes 1, pGL3‐basic; 2, pGL3‐WT; 3, pGL3‐11271281T; 4, pGL3‐11271431T; 5, pGL3‐11271467T; 6, pGL3‐11271576C; 7, pGL3‐11271763A; 8, pGL3‐11272004G; 9, pGL3‐11272071A; 10, pGL3‐11272171T; 11, pGL3‐11272227G>T; 12, pGL3‐11272338A; and 13, pGL3‐11272355A. (b) Relative activities of wild‐type and variant *ATG7* gene promoters in H9c2 cells. Lanes 1, pGL3‐basic; 2, pGL3‐WT; 3, pGL3‐11271281T; 4, pGL3‐11271467T; 5, pGL3‐11271763A; 6, pGL3‐11272004G; 7, pGL3‐11272071A; 8, pGL3‐11272171T; 9, pGL3‐11272338A; and 10, pGL3‐11272355A. WT, wild type. *, *p* < 0.05; **, *p* < 0.01

In HEK‐293 cells, the DSV (g.11271467C>T) and the SNPs [g.11271763G>A (rs76708041), g.11272004C>G (rs550744886), g.11272071G>A (rs544484285), and g.11272355A>G (rs2606729)] identified in AMI patients significantly decreased the transcriptional activity of the *ATG7* gene promoter (*p* < 0.01). In contrast, the DSV (g.11272338G>A) and the SNP [g.11271281T>C (rs2594975)] identified in AMI patients significantly increased the transcriptional activity of the *ATG7* gene promoter (*p* < 0.01). As expected, the DSVs (g.11271319G>C, g.11271431C>T, g.11271576G>C, and g.11272227G>T) identified in controls and the SNP [g.11272171G>T (rs140788185)] found in both AMI patients and controls did not affect the activity of the *ATG7* gene promoter (*p* > 0.05; Figure 2a).

To further determine the tissue‐specific effects of the DSVs and SNPs in cardiomyocytes, the activity of wild‐type and variant *ATG7* gene promoters were examined in H9c2 cells. All the DSVs and SNPs found in AMI patients, except one SNP, exhibited similar effects on the transcriptional activity of *ATG7* gene (*p* < 0.05; Figure 2b). The SNP [g.11272355A>G (rs2606729)] did not significantly affect the transcriptional activity of *ATG7* gene promoter in H9c2 cells, indicating its tissue‐specific effect (*p* > 0.05). Therefore, the DSVs and SNPs identified in AMI patients significantly altered the activity of the *ATG7* gene promoter in both HEK‐293 and H9c2 cells.

### 3.4 The binding for transcription factors affected by the DSVs

**Figure 3:**
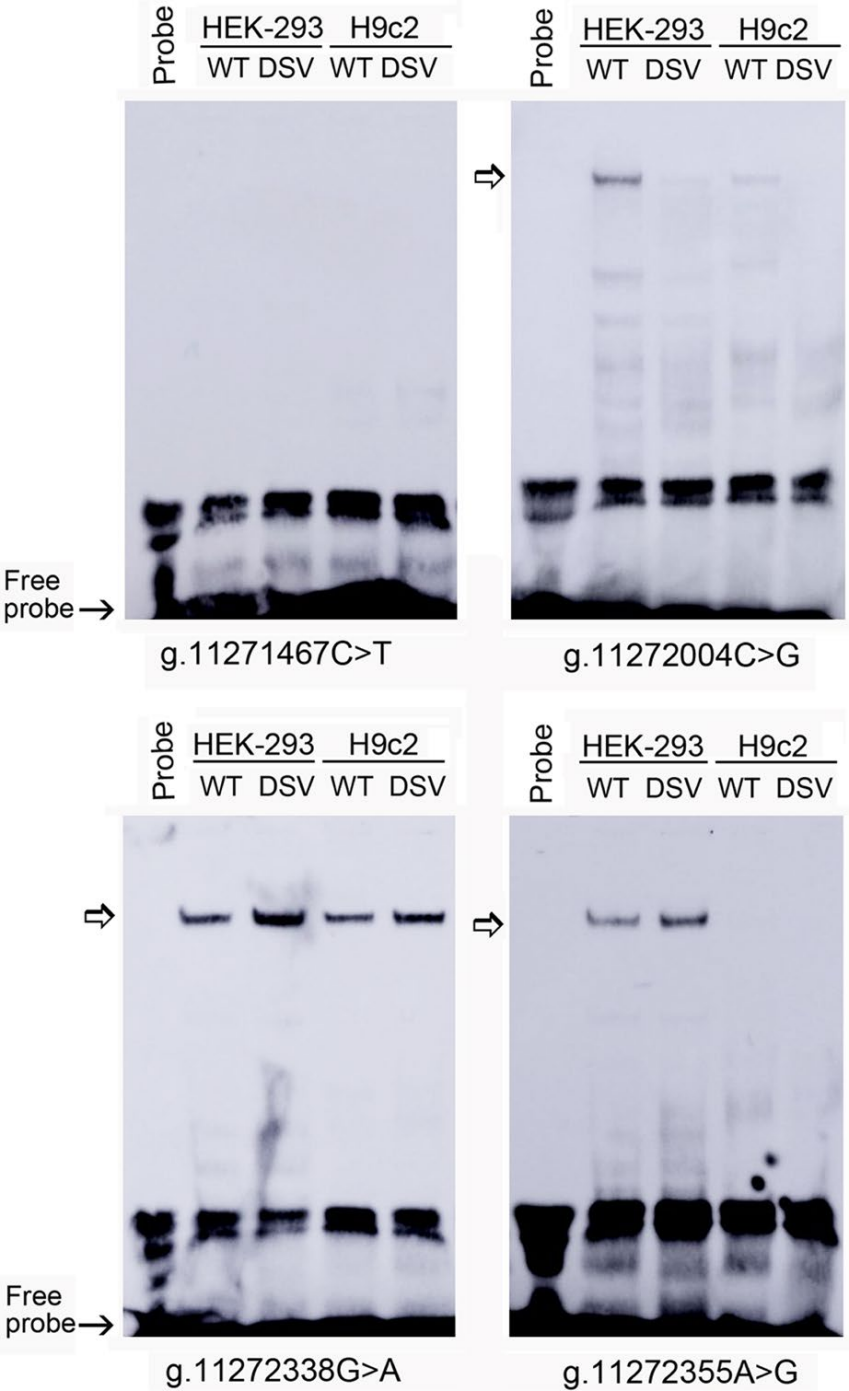
EMSA of biotin‐labeled oligonucleotides containing DSVs and SNPs. Wild‐type and variant oligonucleotides (30 bp) were designed and labeled with biotin for the DSVs and SNPs identified in AMI patients, including g.11271467C>T, g.11272004C>G, g.11272338G>A, and g.11272355A>G. EMSA was conducted with biotinylated oligonucleotides and the nuclear extracts from HEK‐293 and H9c2 cells. Free probe was marked with an arrow at the bottom. The affected binding for transcription factors was marked with an open arrow

To investigate whether the DSVs and SNPs affected the binding for transcription factors, EMSA was performed with wild‐type or variant oligonucleotides. The sequences of the DSVs and SNPs are shown in Table 4. The results showed that the SNP [g.11272004C>G (rs550744886)] reduced the binding of a transcription factor, which was weakly expressed in H9c2 cells. The predicted creation of RHOXF1 binding site may not exist or beyond the detection of EMSA. The DSV g.11272338G>A enhanced the binding of a transcription factor. The SNP [g.11272355A>G (rs2606729)] enhanced the binding of a transcription factor in HEK‐293 cells (Figure 3). Interestingly, this SNP [g.11272355A>G (rs2606729)] did significantly decrease the transcriptional activity of the *ATG7* gene promoter in HEK‐293 cells, but had no effect in H9c2 cells, indicating its tissue‐specific effects (Figure 2a,b). The DSV g.11271467C>T did not affect the binding of transcription factors, likely due to the sensitivity of EMSA (Figure 3). Similarly, other DSVs and SNPs found in AMI patients did not evidently affect the binding of transcription factors (not shown). Taken together, the DSVs and SNPs altered *ATG7* gene promoter activity by affecting the binding of transcription factors. Further work will be needed to identify these transcription factors.

**Table 4:** The double‐stranded biotinylated oligonucleotides for the EMSA

DSVs	Oligonucleotide sequences	Locations
g.11271281T>C (rs2594975)	5′‐GCTGGAGTGCAGTGG(T/C)GGGATCTCGGCT‐3′	11271266‐11271295
g.11271467C>T	5′‐ACCTCGTGATCCGCC(C/T)GCCTCGGCCTCCCA‐3′	11271452‐11271481
g.11271763G>A (rs76708041)	5′‐TTAATAGCAGTCATC(G/A)CTCTTGTTGTTATG‐3′	11271748‐11271777
g.11272004C>G (rs550744886)	5′‐GTCGACGTTCACTGG(C/G)CTTTTCCTACTAAA‐3′	11271989‐11272018
g.11272071G>A (rs544484285)	5′‐TGGCCCCTGTGCTGC(G/A)TTTGATGCCGCCTC‐3′	11272056‐11272085
g.11272338G>A	5′‐CCTTTGCGCACGCGC(G/A)CCGCTTCCCAGTGG‐3′	11272323‐11272352
g.11272355A>G (rs2606729)	5′‐CGCTTCCCAGTGGCA(A/G)GCGCGGGCAGGACC‐3′	11272340‐11272369

## 4 DISCUSSION

Recently, low‐frequency and rare genetic variants have been suggested to account for the missing heritability as a major cause for human diseases (Lettre, 2014). Previous studies have shown that the polymorphism (V471A) in *ATG7* gene that substitutes alanine for valine has been significantly associated with an earlier onset of Huntington's disease (HD) (Metzger et al., 2010). An intron variant in *ATG7* gene (rs8154) is significantly associated with breast cancer‐specific survival (Zhou et al., 2017). Lower and elevated ATG7 levels have been observed in the patients with ulcerative colitis and active eosinophilic esophagitis, respectively (Lankarani, Sepehrimanesh, Seghatoleslam, Hoseini, & Ghavami, 2017; Merves et al., 2016). In this study, we identified two novel DSVs and five SNPs in AMI patients, which significantly altered the transcriptional activity of *ATG7* gene. Further EMSA revealed that one DSV and two SNPs evidently affected the binding of transcription factors. Collective frequency of the DSVs and SNPs in *ATG7* gene promoter was 2.54% (9/355) in AMI patients. Therefore, these DSVs and SNPs may change ATG7 levels, contributing to the AMI development as a low‐frequency risk factor.

The human *ATG7* gene has been mapped to chromosome 3p25.3 and is ubiquitously expressed in human adult and fetal tissues (Tanida et al., 2002). The promoter of the *ATG7* gene has not been characterized in details. Heat shock factor 1 (HSF1), a master regulator of heat shock responses, directly binds to the *ATG7* gene promoter and transcriptionally upregulating its expression (Desai et al., 2013). *ATG7* gene has been validated as target of microRNA‐17 family (microRNA‐20a and microRNA‐17), which bind to the 3′ UTR of *ATG7* gene mRNA (Guo, Mei, Li, Huang, & Yang, 2016). In HeLa cells, p300 acetyltransferase colocalizes and physically interacts with ATG7 in regulating autophagy (Lee & Finkel, 2009). In human umbilical vein endothelial cells, ATG7 interacts with acetylated FOXO1, a mediator of autophagy, in protecting cell survival from oxidative stress damage (Han et al., 2012). Therefore, the DSVs in the *ATG7* gene promoter may change ATG7 levels.

Accumulating evidence suggests that ATG7 and autophagy play protective roles in cardiomyocytes, vascular endothelial cells, and smooth muscle cells (Michiels et al., 2015; Pattison et al., 2011; Torisu et al., 2016). Autophagy is an innate and potent process that protects cardiomyocytes from ischemic death during acute myocardial infarction in animal experiments (Orogo & Gustafsson, 2015). Autophagy also occurs in advanced atherosclerotic plaques and is protective for plaque stabilization (Schrijvers, De Meyer, & Martinet, 2011). In human atrial myofibroblasts, *ATG7* gene knockdown decreases the fibrotic effect of transforming growth factor‐β1 (TGF‐β1) (Ghavami et al., 2015). In contrast, excessive autophagy is capable of provoking plaque destabilization and lesional thrombosis, leading finally to autophagic cell death (Martinet & De Meyer, 2009). Autophagy acts as a mechanism of cell death in atherosclerotic vascular smooth muscle cells (VSMCs) (Jia, Cheng, Gangahar, & Agrawal, 2006). Overloading autophagy has been recognized as a deleterious process by excessive self‐digestion (Tai, Hu, Peng, Zhou, & Zheng, 2016). Endothelial‐specific deletion of *ATG7* gene impairs the release of von Willebrand factor, which plays a major role in blood coagulation (Torisu et al., 2013). Taken together, the DSVs in *ATG7* gene identified in AMI patients may increase or decrease ATG7 levels, which lead to defective or excessive autophagic activity, contributing to the development of atherosclerosis and AMI.

Non‐autophagic functions of ATG7 have been reported. In mouse embryonic fibroblasts, ATG7 could bind to the tumor suppressor p53 to regulate the expression of the cell cycle inhibitor p21(CDKN1A) gene, which is independent of its E1‐like enzymatic activity (Lee et al., 2012). ATG7 functions as cellular scaffolds in regulating phosphorylation of extracellular signal‐regulated kinase (ERK), which controls various aspects of cell physiology including proliferation (Martinez‐Lopez, Athonvarangkul, Mishall, Sahu, & Singh, 2013). ATG7 deficiency suppresses apoptosis and cell death induced by lysosomal photodamage (Kessel, Price, & Reiners, 2012). ATG7 directly interacts with caspase‐9 and represses its apoptotic activity, which is not related to its autophagic function (Han et al., 2014). ATG7 is involved in a process designated LC3‐associated phagocytosis, which is independent of autophagic process (Sanjuan et al., 2007). In addition, ATG7 is required for interferon gamma‐mediated host defense against virus replication (Hwang et al., 2012). Therefore, deficient or excessive ATG7 may interfere with inflammation, cell proliferation, and apoptosis, contributing to the development of cardiovascular diseases through its non‐autophagic function.

In conclusion, the *ATG7* gene promoter was genetically and functionally analyzed in AMI patients and healthy controls in this study. The DSVs and SNPs were identified in AMI patients, which significantly altered the transcriptional activity of the *ATG7* gene promoter. These DSV and two SNPs evidently affected the binding of transcription factors. Therefore, these DSVs and SNPs may change ATG7 level, contributing to AMI development through its autophagic and non‐autophagic functions as a low‐frequency risk factor.

## CONFLICT OF INTEREST

The authors declare that they have no conflict of interest.
